# Severe influenza A(H1N1)pdm09 in pregnant women and neonatal outcomes, State of Sao Paulo, Brazil, 2009

**DOI:** 10.1371/journal.pone.0194392

**Published:** 2018-03-26

**Authors:** Ana Freitas Ribeiro, Alessandra Cristina Guedes Pellini, Beatriz Yuko Kitagawa, Daniel Marques, Geraldine Madalosso, Joao Fred, Ricardo Kerti Mangabeira Albernaz, Telma Regina Marques Pinto Carvalhanas, Dirce Maria Trevisan Zanetta

**Affiliations:** 1 Epidemiological Surveillance Center, Sao Paulo State Secretary of Health, São Paulo, São Paulo, Brazil; 2 Department of Epidemiology, School of Public Health - University of Sao Paulo, São Paulo, São Paulo, Brazil; Defense Threat Reduction Agency, UNITED STATES

## Abstract

To investigate the factors associated with death and describe the gestational outcomes in pregnant women with influenza A(H1N1)pdm09, we conducted a case-control study (deaths and recovered) in hospitalized pregnant women with laboratory-confirmed influenza A(H1N1)pdm09 with severe acute respiratory illness (SARI) in the state of São Paulo from June 9 to December 1, 2009. All cases were evaluated, and four controls that were matched by the epidemiological week of hospitalization of the case were randomly selected for each case. Cases and controls were selected from the National Disease Notification System-SINAN Influenza-web. The hospital records from 126 hospitals were evaluated, and home interviews were conducted using standardized forms. A total of 48 cases and 185 controls were investigated. Having had a previous health visit to a healthcare provider for an influenza episode before hospital admission was a risk factor for death (adjusted OR (*OR*_*adj*_*)* of 7.93, 95% CI 2.19–28.69). Although not significant in the multiple analysis (*OR*_*adj*_ of 2.13, 95% CI 0.91–5.00), the 3^rd^ trimester deserves attention, with an OR = 2.22, 95% CI 1.13–4.37 in the univariate analysis. Antiviral treatment was a protective factor when administered within 48 hours of symptom onset (*OR*_*adj*_ = 0.16, 95% CI 0.05–0.50) and from 48 to 72 hours (*OR*_*adj*_ = 0.09, 95% CI 0.01–0.87). There was a higher proportion of fetal deaths and preterm births among cases (p = 0.001) and live births with low weight (p = 0.019), compared to control subjects who gave birth during hospitalization. After discharge, control subjects had a favorable neonatal outcome. Early antiviral treatment during the presence of a flu-like illness is an important factor in reducing mortality from influenza in pregnant women and unfavorable neonatal outcomes. It is important to monitor pregnant women, particularly in the 3^rd^ trimester of gestation, with influenza illness for diagnosis and early treatment.

## Introduction

Pregnancy constitutes an important risk for the development of influenza-related complications and hospitalization. The 1918 and 1957 influenza pandemics showed increased mortality in pregnant women [1,2]. The reasons for the increased risk during pregnancy probably derive from a combination of immunological and physiological factors. An increased susceptibility to certain intracellular pathogens has been described [3].

The identification of a new viral subtype, influenza A(H1N1)pdm09, in Mexico and the United States in April 2009 and its worldwide dissemination led the WHO to announce the beginning of a pandemic in June 2009 [4]. A study developed during the first month of the outbreak in United States estimated that the rate of admission for pandemic H1N1 influenza in pregnant women was higher than in the general population (0.32 per 100,000 pregnant women, 95% CI 0.13–0.52 vs 0.076 per 100,000 population at risk, 95% CI 0.07–0.09)[5]. Since 2012, the World Health Organization (WHO) has defined this group as the highest priority for vaccination [6].

A study conducted in California among hospitalized pregnant women and women of reproductive age with influenza A(H1N1)pdm09 showed that pregnant women in the 2^nd^ and 3^rd^ trimesters who delayed treatment(≥ 48 hours) were more likely to undergo admission to the ICU or death [7]. Most studies evaluate risk factors for increased severity in pregnant women [8,9,10,11] and few studies have analyzed the risk factors for death in pregnant women from influenza A(H1N1)pdm09. A systematic review study showed a higher risk of hospitalization in pregnant women with influenza A(H1N1)pdm09, with relative risks ranging from 4.3 to 7.2. However, only one study showed a higher risk of death in pregnant women with influenza A (H1N1)pdm09 (RR 10.2) and seven studies presented no significant risks (0.3 to 1.3)[12]. Meta-analysis also showed no higher risk of death in pregnant women with pandemic influenza, OR 0.99 (95% CI 0.67 to 1.46)[8]. A higher risk of fetal abnormalities in pregnant women with both seasonal influenza virus infection and A(H1N1)pdm09 infection was reported. In addition, women with a diagnosis of A(H1N1)pdm09 infection had a higher risk of placental problems, antepartum haemorrhage, and antepartum complications [13].

During the pandemic, there were a significant number of deaths in pregnant women from influenza A(H1N1)pdm09 in São Paulo, which justifies this study. The objective of this study was to analyze factors associated with death in pregnant women with influenza A(H1N1)pdm09 and severe acute respiratory illness (SARI) and describe the gestational and neonatal outcomes.

## Methods

The State of São Paulo has a population of more than 41 million inhabitants, with 598,473 live births in 2009, according to data from the Information Department of the Unified Public Health System (DATASUS) of the Ministry of Health.

A case-control study was conducted that evaluated pregnant women living in São Paulo with confirmed infection of influenza A(H1N1)pdm09 and hospitalized with SARI, defined as: fever and cough and dyspnea or pneumonia or respiratory failure or tachypnea or radiological alterations consistent with pneumonia or oxygen therapy or mechanical ventilation. The definition of SARI has been adapted from the one stated by the WHO to increase sensitivity in the detection of cases and controls [14].

In 2009, the Ministry of Health of Brazil established the compulsory notification of any hospitalized case of influenza associated with SARI and the inclusion of an epidemiological investigation into the Influenza-web database of the National Disease Notification System (SINAN). All hospitalized pregnant women who were notified with influenza associated with SARI and eventually died during the epidemic period from July 9^th^ to December 1^st^ 2009 in São Paulo were included in the analysis. For each death (case), four controls were randomly selected from those who recovered. The cases and controls were identified from the SINAN, using the following variables for selection: RT-PCR (positive for influenza A/H1N1pdm09), final classification (confirmed), evolution (recovered or death), hospitalization (yes), date of hospitalization, hospital and residence in the São Paulo State. The controls were matched by epidemiological week of admission date of the case to adjust for possible variations in access to treatment and clinical protocols. All pregnant women had a laboratory confirmation of influenza A(H1N1)pdm09 from a sample of respiratory secretions using the RT-PCR method, performed at the Adolfo Lutz Institute, the public health laboratory [15].

For data collection, trained health professionals used two standardized forms: one to collect hospital record information and the second was used for home interviews. For the cases, the interviews were conducted with close family members, and for the controls, the interviews were conducted with the patients themselves. The hospital form included the following variables: pathological history, health care, symptoms on admission, admission to the intensive care unit, antiviral treatment (the Ministry of Health released the antiviral oseltamivir), use of antibiotics, complications, laboratory tests, radiological examinations, evolution, gestational and neonatal outcomes. The home form included the following variables: sociodemographics, history of a previous health visit to a healthcare provider for the influenza episode that resulted in hospitalization (after the onset of symptoms and before hospital admission date), vaccination history and gestational and neonatal outcome. Education level was classified as low (no schooling or incomplete primary), medium (complete primary or incomplete high school) or high (complete high school or university). Occupations were grouped using the occupational risk pyramid for the pandemic in the occupational safety and health Act of the National Institute for Occupational Safety and Health-CDC, which classified the occupation risk for influenza infection as low (professional managers and other university and technical professionals without close contact with the population), medium (professionals in the areas of education, trade, service and administration with close contact with the population), or high and very high risk (doctors, nurses, other health professionals and support staff in the health services) [16]. A pre-test with 10 patients with Influenza A(H1N1)pdm09 was performed to identify and correct any errors. The questionnaires are presented in S1 Data collection form and the complete data used in this study are shown in S1 Database.

The study was started during the epidemic to support the actions of epidemiological surveillance, and the use of the data was approved by the Ethics Committee of the School of Public Health of USP (Protocol 2283, OF.COEP/312/11). Data collection complied with the recommendations of the National Health Council for Research in Human Beings, including the signing of a consent form.

Clinical and demographic variables are presented as medians and interquartile ranges or percentages, Mann-Whitney U or chi-square tests were used for comparisons, as appropriate. Odds ratios and 95% confidence intervals (95% CI) were calculated to evaluate factors associated with death.

For the multiple logistic regression, variables were selected with a p of <0.20 in the univariate analysis and those considered important for the adjustment. The initial model included: health plan; previous visit to a healthcare provider; the presence of at least one of the high-risk medical conditions for developing influenza-related complications (adapted from the Centers for Disease Control and Prevention): asthma, neurological and developmental disorders, heart disease, kidney disease, liver disease, hemoglobinopathies, endocrine disorders, immunosuppressive diseases and obesity, with the absence of these conditions as reference; the use of an antiviral [no use (as reference), ≤ 48 hours from the first symptoms, > 48 and ≤ 72 hours, > 72 hours], pregnancy trimester [1^st^ and 2^nd^ trimesters (as reference) and 3^rd^ trimester]. The data were controlled for education level and age. The Wald and Hosmer-Lemeshow tests were used to evaluate the significance of the variables and test the fit of the model, respectively.

Analyses were performed using SPSS (IBM, Armonk, NY, USA), version 17.0. P values of <0.05 were considered significant.

## Results

**Fig 1:**
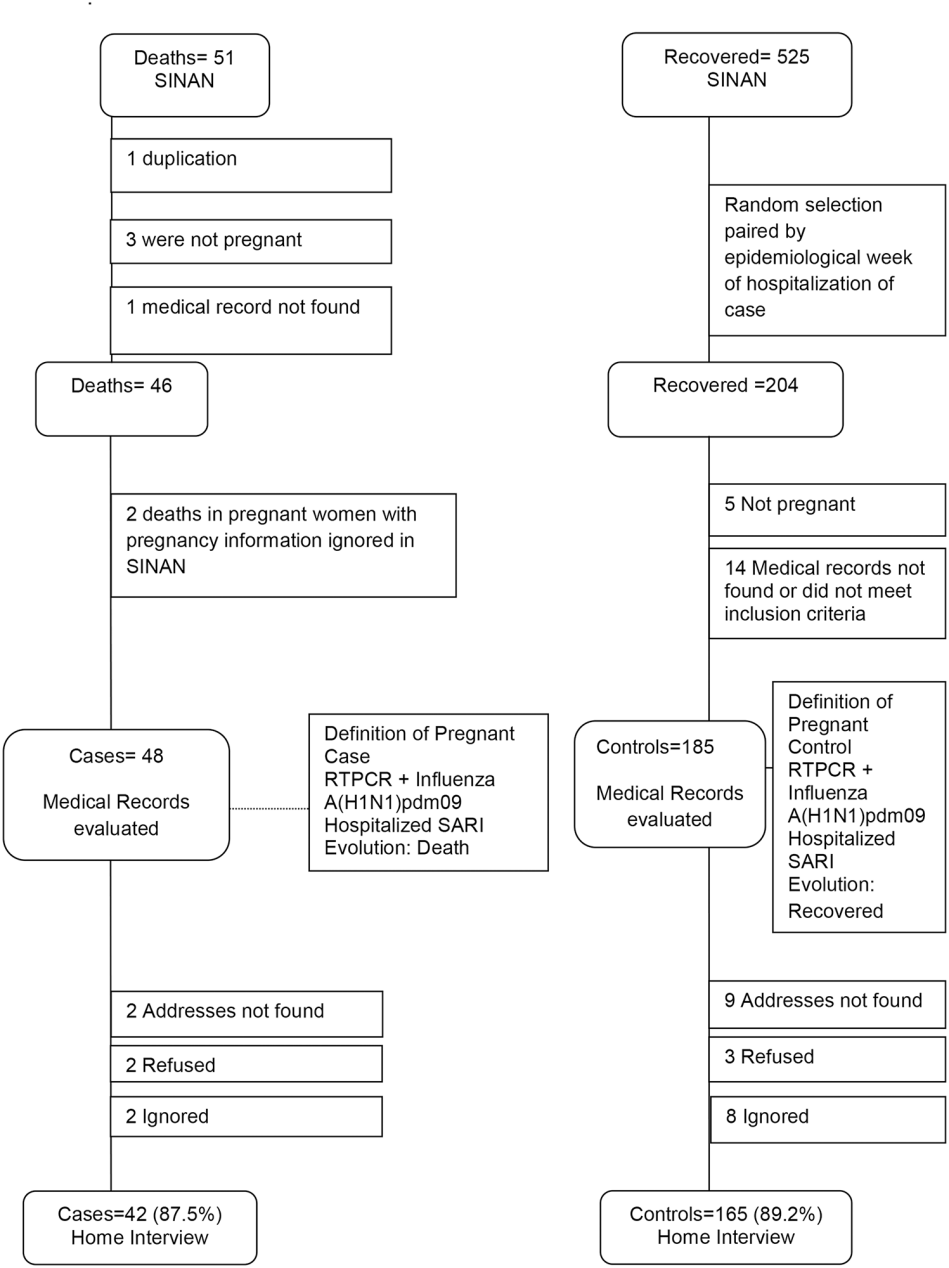
Flow chart for the selection of cases and controls among pregnant women reported in the National Disease Notification System—SINAN—São Paulo State, 2009.

In São Paulo, in 2009, 51 pregnant women with confirmed influenza A(H1N1)pdm09 infection notified in SINAN eventually died. A total of 204 controls were randomly selected among the 525 pregnant women hospitalized with confirmed influenza A(H1N1)pdm09 infection notified in SINAN who recovered. The investigation was conducted in 126 hospitals where all cases and controls were hospitalized. With regard to the 51 cases and 204 controls initially identified in SINAN for the study, 22 files were missing or did not meet the case or control requirements, resulting in 48 cases and 185 controls reviewed. Two pregnant women who died were identified in another study[13], and included in the present. The home interviews were performed for 42 cases (87.5%) and 165 controls (89.2%), as shown in Fig 1.

Table 1 presents the socio-demographic characteristics of the pregnant women. There were no significant differences in socio-demographic distribution between cases and controls, and median age, family income, education level, smoking history, occupational risk for influenza infection, previous pregnancy and existence of private health insurance were not associated with death.

**Table 1:** Distribution of pregnant women hospitalized with influenza A(H1N1)pdm09 associated with severe acute respiratory syndrome, who died (cases) or recovered (control), according to general and sociodemographic characteristics, State of Sao Paulo, 2009.

Characteristics	N	Cases	N	Controls	OR^a^ 95% CI	*p*
		n (%)		n (%)		
Age Group (years)^b^	48		185			
15–19		7 (14.6)		30 (16.2)	1	
20–29		31 (64.6)		112 (60.5)	1.19 (0.48–2.96)	
30–39		10 (20.8)		40 (21.6)	1.07 (0.37–3.14)	
40–49		0 (0.0)		3 (1.6)	.	
Age, median (IQR)^b^^,^^d^		24.71 (21.04–29.35)		24.58 (21.63–29.74)		0.836
Previous Pregnancy^b^	47		184			0.970
0		17 (36.2)		70 (38.0)		
1–4		28 (59.6)		106 (59.6)		
≥ 5		2 (4.2)		8 (4.4)		
Race/Color^c^	41		165			
White		23 (56.1)		91 (55.7)	1	
Black/ Mixed /Yellow		18 (43.9)		74 (44.3)	0.96 (0.48–1.92)	
Private Health Plan^c^	41	15 (36.6)	165	63 (38.2)	0.93 (0.46–1.90)	
Family Income ^c^^,^^e^	40		163			
Up to 02 MS		20 (50.0)		87 (53.4)	1.23 (0.33–4.61)	
02 to 04 MS		14 (35.0)		40 (24.5)	1.87 (0.47–7.38)	
04 to 08 MS		3 (7.5)		20 (12.3)	0.80 (0.14–4.51)	
> 08 MS		3 (7.5)		16 (9.8)	1	
Educational Level^c^^,^^f^	41		165			
Low		7 (17.1)		27 (16.1)	1.44 (0.54–3.87)	
Medium		18 (43.9)		49 (29.7)	2.04 (0.96–4.36)	
High		16 (39.0)		89 (53.9)	1	
Smoker^c^^,^^g^	41	9 (22.0)	165	32 (19.4)	1.17 (0.51–2.69)	
Occupation^c^	41	21 (51.2)	165	87 (52.7)	0.94 (0.47–1.87)	
Occupational Risk^c^^,^^h^						
Very high and high		0 (0.0)		7 (8.1)		
Medium		19 (90.5)		69 (79.3)	1.51 (0.31–7.42)	
Low		2 (9.5)		11 (12.6)	1	

^a^ OR, Crude Odds Ratio, not adjusted

^b^ Data collected from hospital records

^c^ Data collected from home interviews

^d^ IQR—Interquartile range

^e^ One case and two controls ignored, MS Minimum Salary (R$ 465.00) in 2009.

^f^ Low: no schooling or incomplete primary; medium: complete primary or incomplete high school; High: complete high school or university

^g^ Smoked at the time of hospitalization

^h^ Very high and high: doctors, nurses, dentists, other health professionals and support staff in the health services; medium: professionals in the areas of education, trade, service and administration with close contact with the population; low: professional managers and other university and technical professionals without close contact with the population

Table 2 shows the distribution of cases and controls according to clinical variables and visits to a healthcare provider. A total of 92.7% of cases sought medical care for the influenza episode prior to hospitalization. This proportion was higher than that of the controls. When evaluating the preconditions for admission, the presence of other risk conditions for developing complications related to influenza did not differ in cases and controls. Asthma and obesity were the most common conditions, among cases (8.3% each) and controls (8.9% vs. 3.2%, respectively).

**Table 2:** Distribution of pregnant women hospitalized for influenza A(H1N1)pdm09 associated with severe acute respiratory illness who died (cases) or recovered (controls) according to risk conditions and clinical aspects, State of Sao Paulo, 2009.

Characteristics	N	No. (%) cases	N	No. (%) controls	OR^a^95%CI	*P*
Trimester of Pregnancy^b^	48		185			
First/Second		15 (31.2)		93 (50.3)	1	
Third		33 (68.8)		92 (49.7)	2.22 (1.13–4.37)	
Previous visit to healthcare provider^c^	41	38 (92.7)	165	101 (61.2)	8.03 (2.38–27.09)	
Influenza Vaccine 2009^c^^,^^d^	37	0 (0.0)	161	15 (9.3)		
Risk Conditions^b^	48		185			
None		36 (75.0)		149 (80,5)	1	
At least one		12 (25.0)		36 (19.5)	1.38 (0.65–2.91)	
Asthma		4 (8.3)		16 (8.9)		
Obesity^e^		4 (8,3)		6 (3,2)		
Chronic Pulmonary Disease^f^		2 (4.2)		1 (0.5)		
Diabetes Mellitus		0 (0.0)		2 (1.1)		
Immunosuppression^g^		2 (4.2)		2 (1.1)		
Chronic Kidney Disease		0 (0.0)		3 (1.6)		
Chronic Liver Disease		0 (0.0)		2 (1.1)		
Blood disease (hemoglobinopathies)		0 (0.0)		2 (1.1)		
Symptomatology^b^	48		185			
Fever		44 (91.7)		176 (95.1)		0,350
Cough		42 (87.5)		180 (97.3)		0,004
Dyspnea		43 (89.6)		148 (80.0)		0,123
Antiviral Use^b^	48		185			
No		11 (22.9)		16 (8.6)	1	
Yes		37 (77.1)		169 (91.4)	0.32 (0.14–0.74)	
≤ 48 hours of first symptoms		10 (27.0)		107 (63.4)	0.14 (0.05–0.37)	
>48 and ≤ 72 hours of firstsymptoms		2 (5.4)		22 (13.0)	0.13 (0.03–0.68)	
> 72 hours of first symptoms		25 (67.6)		40 (23.7)	0.90 (0.36–2.27)	
Other^b^	48		185			
Intensive Care Unit (Yes)		46 (95.8)		30 (16.2)		< 0,001
Antibiotic use (Yes)		48 (100.0)		120 (64.9)		< 0,001
Ventilator use (Yes)		48 (100.0)		24 (13.0)		< 0,001
Time/Days, median (IQR)^b^^,^^h^						
First symptoms to hospitalization	48	4 (1–6)	185	2 (1–3)		0,003
Hospitalization until discharge/death	48	11.5 (5–15)	185	4 (3–7)		< 0,001
First symptoms until starting antiviral^i^	37	5 (2–7,5)	169	2 (1–3)		< 0,001
Hospitalization until starting antiviral^i^	37	1 (0–3)	169	0 (0–1)		< 0,001

^a^ OR_,_ Crude Odds Ratio

^b^ Data collected from hospital records

^c^ Data collected from home interviews

^d^ 4 Cases ignored and 3 Controls ignored

^e^ Referred to in the medical records

^f^ Chronic pneumonitis, chronic obstructive pulmonary disease, cystic fibrosis, bronchiectasis.

^g^ Malignant neoplasm, autoimmune disease, immunosuppressive drugs, organ transplantation and HIV/Aids

^h^ IQR—Interquartile range

^i^ 37 cases and 169 controls with antiviral treatment

The use of an antiviral during hospitalization was an important protective factor against death, with proportions of treatment at 77.1% and 91.4% in cases and controls, respectively. The proportions of women who received antiviral within the first 48 hours of symptoms were 27% and 63.4% in cases and controls, respectively. The protective effect was also observed for treatment starting within 48 to 72 hours (5.4% and 13% in cases and controls, respectively). The initiation of treatment with an antiviral more than 72 hours after the onset of symptoms did not present significant protection. The median number of days between the date of the first symptoms and hospitalization was four for the cases and two for the controls (p = 0.003). Treatment with antibiotics was used in 100% of cases and 64.9% of controls. The average number of antibiotics used was 3.9 for cases and 1.1 for controls. Among the cases, there was a higher proportion of patients admitted to the intensive care unit (95.8% vs. 16.2%), and a higher proportion of cases underwent mechanical ventilation compared to controls (100% vs. 13%). The cases exhibited higher rates of complications than the controls (100% vs. 10.3%), predominantly: respiratory distress syndrome (72.9% vs. 4.9%), shock (75.0% vs. 0.5%), sepsis (64.6% vs. 3.2%), infections (35.4% vs. 3.8%) and renal alterations (35.4% vs. 2.7%). There were three episodes of pre-eclampsia, which evolved to death (cases) and one in the control group. None of them fulfilled the definition of HELLP syndrome, named for three features of the disease (hemolysis, elevated liver enzyme levels, and low platelet levels). All cases that evolve to death had influenza A(H1N1)pdm09 as the main cause in the death certificate.

Co-infection with other infectious agents occurred in 20.4% of cases and 1.6% of controls, with the following pathogens found: *Acinetobacter baumannii* (n = 4), *Klebsiella pneumoniae* (n = 3), *Staphylococcus aureus* (n = 3), *Pseudomonas aeruginosa* (n = 2), Enterococcus spp. (n = 2), *Candida* spp (n = 2), *Candida albicans* (n = 1), *Klebsiella* spp. (n = 1) and *Streptococcus pneumoniae* (n = 1). The results of chest radiology were assessed in 93.8% of cases and in 80% of controls. Among the cases, 91.6% presented with alterations, with a consolidation pattern evident in 50% of cases. Among the controls, 59.5% presented alterations, with a consolidation pattern evident in 14.4% of cases.

Table 3 shows the findings from the laboratory examinations at the time of hospital admission. The cases presented lower median platelet, hemoglobin and hematocrit counts and higher median levels of creatine phosphokinase-CPK, lactate dehydrogenase-LDH, glutamic oxaloacetic transaminase-GOT, urea and creatinine, with statistical significance.

**Table 3:** Laboratory examinations of pregnant women hospitalized with influenza A(H1N1)pdm09 associated with severe acute respiratory illness, who died (cases) or recovered (controls), State of São Paulo, 2009.

Laboratory Examinations^a^^,^^b^	n	Cases N = 48	N	Controls N = 185	
		Median (IQR)^c^		Median (IQR)^c^	*p*
Hemoglobin (g/dL)	46	10.6 (9.9–11.5)	164	11.4 (10.5–12.2)	*0*.*001*
Hematocrit (%)	46	32.7 (29.4–34.7)	163	34.1 (31.4–36.0)	*0*.*005*
Leukocytes (cel/mm^3^)	46	8500 (6125–11075)	154	8200 (6575–10377.5)	*0*.*947*
Platelets (u/L)	47	160000 (139000–204000)	159	200000 (161000–239000)	*<0*.*001*
Creatine phosphokinase—CPK(U/L)	12	205 (94.5–623.75)	12	53.5 (39.5–79.75)	*0*.*006*
Lactate dehydrogenase-LDH (U/L)	16	606.5 (411.5–875.5)	29	279 (173–610.5)	*0*.*009*
Glutamic oxaloacetic transaminase-GOT(U/L)	33	67 (46–102.40)	38	26.5 (21.75–53.50)	*<0*.*001*
Glutamic pyruvic transaminase-GPT(U/L)	32	35 (25.25–42.33)	38	24.50 (16.75–40.25)	*0*.*056*
Urea (mg/dl)	43	17 (15–24)	84	14 (11–19.75)	*0*.*017*
Creatinine (mg/dl)	43	0.70 (0.50–0.90)	92	0.60 (0.46–0.70)	*0*.*003*

^a^ First hospital examination

^b^ Data collected from hospital records

^c^ IQR—Interquartile range

Table 4 presents the variables in the final multiple logistic regression model. Having had a previous health visit to a healthcare provider for the influenza episode before hospitalization was a risk factor for death, OR 7.93 (95% CI 2.19–28.69). Antiviral treatment was a protective factor for death when administered within the first 48 hours after the onset of symptoms, OR 0.16 (95% CI 0.05–0.50), and when administered 48 to 72 hours after the onset of symptoms, OR 0.09 (95% CI 0.01–0.87). The third trimester of gestation, which was a significant risk factor in univariate analysis, lost significance in the multiple analysis, OR 2.13 (95% CI 0.91–5.00), when antiviral treatment was included in the model. The proportion of women who did not receive any antiviral treatment was similar in the three trimesters of gestation (11.1, 11.1 and 12% respectively) and the proportion of those who received the treatment after 72 hours was higher in the 3^rd^ trimester (14.8%, 25.9% and 32%, respectively for the 1^st^, 2^nd^ and 3^rd^ trimester of gestation.

**Table 4:** Risk factors for death among pregnant women hospitalized with influenza A(H1N1)pdm09, State of Sao Paulo, 2009.

Characteristics	OR^a^ (95% CI)	Adjusted OR_adj_^b^ (95% CI)
Pregnancy trimester		
First/Second	1	1
Third	2.22 (1.13–4.37)	2.13 (0.91–5.00)
Risk conditions		
Presence of at least one^c^	1.38 (0.65–2.91)	1.28 (0.50–3.30)
Previous visit to healthcare provider^c^	8.03 (2.38–27.09)	7.93 (2.19–28.69)
Private health plan^c^	0.93 (0.46–1.90)	1.07 (0.45–2.54)
Antiviral use		
No use	1	1
≤ 48 hours of the first symptoms	0.14 (0.05–0.37)	0.16 (0.05–0.50)
>48 ≤ 72 hours of the first symptoms	0.13 (0.03–0.68)	0.09 (0,01–0.87)
> 72 hours of the first symptoms	0.90 (0.36–2.27)	0.85 (0.29–2.54)

^a^ OR_,_ Crude Odds Ratio

^b^ final model of multiple logistic regression, odds ratio adjusted (OR_adj_) by age and educational level. Hosmer Lemeshow Test 0.662

^c^ the absence of risk conditions was used as reference

As shown in Table 5, among the cases, 45.8% of pregnant women had live births, with one twin birth, and 54.1% of the women experienced fetal deaths, of which fetal deaths later (≥ 23 weeks) represented 65.4% of the total number of deaths. Among the controls, 13.5% delivered during hospitalization, with one twin birth. Regarding the neonatal outcome in this group, there were 7.7% fetal deaths and 92.3% live births. Considering the live births that occurred during hospitalization, in 100% of cases and in 75.0% of controls a cesarean delivery was performed. The distribution of gestational outcomes shows a concentration of miscarriages and premature births among cases compared to controls who delivered during hospitalization: 82.6% and 45.8%, respectively (p = 0.001). Among the 144 controls who were discharged before delivery and who completed a home interview, 100% had live births, 62.5% by cesarean delivery and 86.8% at term.

**Table 5:** Distribution of pregnancy outcomes of pregnant women hospitalized with influenza A(H1N1)pdm09 associated with severe acute respiratory illness who died (cases) or recovered (controls), State of São Paulo, 2009.

Gestational Outcome	Cases			Controls—delivery during hospitalization				Controls—delivery after discharge		
(Weeks)	n (%)			n (%)				n %		
	FD^a^^,^	LB^b^^,^	Tota	FD^a^	LB^b^^,^^c^	Total	*P*^d^	FD^a^	LB^b^^,^^c^	Total
Miscarried^e^ (≤ 22 weeks)	7 (26.9)	0	7 (14.3)	1 (50.0)	0	1 (3.8)		0	0	0
Premature birth^e^ (23 to 36 weeks)	15 (57.7)	19(82.6)	34 (69.4)	0	11 (45.8)	11(42.4)		0	19 13.2	19(13.2)
Full term birth(≥ 37 weeks)	2 (7.7)	4 (17.4)	6(12.2)	0	13 (54.2)	13(50.0)	0.001	0	125 (86.8)	125 (86.8)
Ignored	2 (7.7)	0	2 (4.1)	1 (50.0)	0	1 (3.8)		0	0	0
Total	26 (100.0)	23 (100.0)	49 (100.0)	2 (100.0)	24 (100.0)	26 (100.0)		0	144 (100.0)	144(100.0)

^a^ FD—Fetal death

^b^ LB—Live birth

^c^ Three twins birth (case, control delivery during hospitalization and control delivery after discharge)

^d^ Comparison between controls who delivered during hospitalization vs. cases (Total)—Chi-square

^e^ Grouped for the chi-square calculation

Regarding the live births, among the cases, there was predominance of gestational age at birth between the 32^nd^ and 36^th^ weeks of pregnancy (65.2%), and among the controls who delivered during hospitalization, 54.2% occurred at 37 weeks and over (p = 0.003). Among the controls that gave birth after hospital discharge, 86.8% of births were full term (≥37^th^ week). Analyzing the weight of live births, there was a higher proportion of low birthweight (<2,500 g) among cases (73.9%) than among controls who gave birth during hospitalization (37.5%), p = 0.011. Considering control women who delivered after discharge, low birthweight occurred in only 6.3% of the births. During hospitalization, 8.7% (2 in 23; 28 and 38 days after birth) of the live births of cases and 4.2% (1 in 24; 12 days after birth) of live births of controls evolved to death after giving birth. None of the live births of control women who delivered after discharge evolved to death. The median gestational age, birth weight and Apgar score in the 1^st^ minute were significantly lower among cases than among controls who delivered during hospitalization. Among the cases, 73.7% of the newborns were admitted to the intensive care unit (ICU), contrasting to only 35.0% of newborns from controls who delivered during the hospitalization. When the weight of the newborns was compared with the gestational age, 27.3% of newborns from cases were classified as small for the gestational age, and 12.5% and 7.5% of those from controls who gave birth during and after hospitalization, respectively, as shown in Table 6.

**Table 6:** Distribution of neonatal outcomes (live birth) of pregnant women hospitalized with influenza A(H1N1)pdm09 associated with severe acute respiratory illness who died (cases) or recovered (controls) according to weight, gestational age and Apgar, State of São Paulo, 2009.

Gestational Age	N	Cases^a^^,^^b^	N	Controls^a^^,^^b^- delivery during hospitalization		N	Controls^a^^,^^b^—delivery after discharge
	23	n (%)	24	n (%)	*P*^c^	144	n (%)
	23		24			144	
< 28 weeks^d^		1 (4.3)		2 (8.3)			1 (0.7)
28–31 weeks^d^		4 (17.4)		3 (12.5)			6 (4.2)
32–36 weeks^d^		15 (65.2)		6 (25.0)			12 (8.3)
> = 37 weeks		3 (13.1)		13 (54.2)	0.003		125 (86.8)
Birth weight (grams)	23		24			144	
< 1500		2 (8.7)		3 (12.5)			4 (2.8)
1500–2499		15 (65.2)		6 (25.0)			7 (4.9)
> = 2500		6 (26.1)		15 (62.5)	0.019		133 (92.3)
Birth weight Median (IQR)		2,100 (1,730–2,565)		2,740 (2,229–3,084)	0.015		3,015 (2,736–3,454)
APGR 1^st^ Minute^e^ Median (IQR)	18	3.5 (1.75–8)	21	9 (8–9)	0.001	82	9 (8–9)
APGAR 5^th^ Minute^f^ Median (IQR)	16	8 (3.5–9)	22	9 (8.7–10)	0.003	86	9 (9–10)
SGA^g^	22	6 (27.3)	24	3 (12.5)		133	10 (7.5)
Intensive Care Unit	19	14 (73.7)	20	7 (35.0)		-	-

^a^ 2 live births of cases evolved to death and 1 live birth of control evolved to death12 days after delivery (0.885 grams)

^b^ Three twins birth (case, control delivery during hospitalization and control delivery after discharge)

^c^ Comparison between controls whose live births were delivered during hospitalization vs. cases—Chi-square

^d^ Grouped for the chi-square calculation

^e^ 5 cases of live births with ignored Apgar 1; 3 controls with live births with skipped Apgar1 (delivery during hospitalization) and 62 controls with live births with skipped Apgar1 (delivery after discharge).

^f^ 7 cases of live births with ignored Apgar 5; 2 controls with live births with ignored Apgar 5 and 58 with live births with ignored Apgar 5 (delivery after discharge).

^g^ Small for gestational age (Intergrowth 21)

## Discussion

The case control design, including all reported deaths of hospitalized patients who presented with laboratory confirmed influenza A(H1N1)pdm09 with SARI in the State of São Paulo, and the random selection of four controls for each case, with the collection of hospital data and home interviews, allowed for the expansion of the analysis of risk factors for death. It was possible to evaluate the gestational and neonatal outcomes, including those of pregnant women who delivered after hospital discharge. The main results suggest that an early search for care, the training of physicians for the proper treatment of pregnant women, and early antiviral administration can be protective factors against death. Another noteworthy result was the presence of unfavorable neonatal outcomes, with a significant proportion of stillbirths and miscarriages, low birth weights and lower Apgar scores among pregnant women who died. After hospital discharge, the patients had a favorable neonatal outcome. Pre-eclampsia was present in six percent of women who evolved to death and in less than one percent of those who survived. Despite the fact that all women who died had the influenza A (H1N1)pdm09 infection as the main cause of death in their death certificate, we cannot rule out the possibility that pre-eclampsia may have contributed to their unfavourable outcome.

Age distribution and race were not associated with a risk of death in pregnant women, similar to results in other studies[9,10,11]. The third trimester of gestation lost its significance as risk factor for death in the multiple analyses, when antivirus treatment was included in the model. A higher proportion of women in the final period of gestation received the treatment after 72 hours of symptoms than those who were at earlier stages of gestation. A meta-analysis study showed that the third trimester of gestation was a risk factor for death (OR 1.22, 95% CI 1.01–1.48) for pandemic influenza, when compared with those in the first or second trimester [12]. Although the present study did not confirm the association with the third trimester of gestation, its result indicates that it is important to monitor pregnant women with influenza illness, with special attention during this trimester, for diagnosis and early treatment.

The pregnant women who had a previous health visit to a healthcare provider for the influenza episode before hospitalization had a higher risk of death. This increased risk could be an indication of difficulties in accessing hospitalization or lack of perception of the severity of the case by doctors or lack of recognition that, even in cases that are not serious, considering that pregnant women are in a high risk group for severity of the disease, early antiviral therapy should have been introduced. The median time between the first symptoms and hospitalization was twice as high among pregnant women who died. Similar results were found when patients in general with influenza during the pandemic were evaluated in São Paulo[17] and in Mexico [18]. These findings also reinforce the need for pregnant women to have access to health services, particularly hospitalization in serious cases. The training of physicians concerning the proper care for pregnant women and the need to start early treatment are as important as the early search for care.

The use of an antiviral medication was a protective factor death when administered within 72 hours of symptom onset. Several studies have shown an increased risk of death or worsening disease in pregnant women who started treatment late [10,11,19,20,21,22,23] and in patients in general [17,18,24,25,26,27,28,29].

Regarding the history of previous diseases, no differences in the presence of risk conditions for developing influenza-related complications were found between pregnant women who eventually died and those who recovered. However, surveillance showed a higher proportion of risk conditions among pregnant women who eventually died compared to those who survived [10]. A study in China showed that obesity (BMI ≥ 30) was a factor associated with mortality in patients with severe disease [9]. This result is in line with the current study. Although it was not possible to calculate BMI, a higher rate of obesity among the cases than among the controls was reported.

There were a higher proportion of cases with co-infections than controls, consistent with other reported results [17].

The patients who eventually died presented significant alterations in laboratory values when compared to controls. The alterations observed in CPK, LDH, platelets and creatinine were similar to those reported for patients in general [27,29,30,31,32]. In pregnant women, there was also a decrease in the number of red blood cells.

In relation to neonatal outcomes, there were a greater proportion of fetal deaths in patients who died than in controls that delivered either during hospitalization or after discharge. Among the live births in the cases, there was a greater proportion of low birth weight, gestational age less than 32 weeks, admitted to ICU, lower scores on the Apgar scale, when compared to the live births of controls that delivered during or after hospitalization. Similar results were reported in studies that evaluated pregnant women infected with influenza A(H1N1)pdm09 compared to women of childbearing age without infection[33], pregnant women with influenza A(H1N1)pdm09 with severe disease [9,20] or women who gave birth to live newborns during hospitalization [10,34]. Control women who delivered after discharge from the hospital had favorable outcomes in their offspring, with a median APGAR of 9 both in the 1^st^ and 5^th^ minutes of life. Considering all births in the State of São Paulo during 2009, according to the national system of live births-SINASC, 58% were delivered by cesarean, 9% were preterm birth (<37 weeks) and 9% had low birthweight (≤2500 g). In the control women of the study who gave birth after hospitalization, these proportions were also high (62.5%, 13.2% and 7.7%, respectively).

This study has limitations. Underreporting of SARI by health professionals as well as gaps in the SINAN may have occurred. The notification of influenza through a new viral subtype associated with SARI was initiated during the pandemic; consequently, the sensitivity may have varied with time. The quality of information from medical records can differ between hospitals. The use of standardized hospital and home questionnaires minimized these difficulties during data collection. Although all hospital reports were reviewed, we were not able to establish the roles of other factors, such as secondary infections or obstetric factors, which could have contributed to death in the cases. The results presented in this study indicate that early treatment can prevent unfavorable outcomes in pregnant women and in their offspring and reinforce the need for the proper training of doctors for the clinical management of pregnant women and early administration of antiviral treatment. These findings also support interventions in situations of future pandemics and seasonal influenza with the goals of preventive measures and the organization of health services for the appropriate clinical management of pregnant women.

## Supporting information

S1 Data Collection Form (DOC)

S1 Database (XLSX)
