# Supplementary material for: Severe influenza A(H1N1)pdm09 in pregnant women and neonatal outcomes, State of Sao Paulo, Brazil, 2009
Source: PLoS One. 2018 Mar 26;13(3):e0194392. doi: 10.1371/journal.pone.0194392 (PMC5868799; doi:10.1371/journal.pone.0194392)
Supplement: S1 Data Collection Form — (DOC) [file pone.0194392.s001.doc]

| **EVALUATION OF RISK FACTORS FOR INFLUENZA A (H1N1) NEW VIRAL SUBTYPE, GREATER SÃO PAULO AND MUNICIPALITIES OF GVE CAMPINAS, 2009.** |
| --- |

| **PART II - HOSPITAL** |
| --- |

| **Identification Number:** | **____________** |
| --- | --- |

| **Classification:** | **1 – Case** | 2- **Control** |  |
| --- | --- | --- | --- |
| **Identification of pairing (to be completed by monitor):** | | |  |
| **Evolution:** | **1 – Death** | 2- C**ure** |  |

| **Interviewer:** | **________________________________________** | **______ ______** |
| --- | --- | --- |
| **Date:** | __________/__________/2010 | |

| **Patient Name:** |  |
| --- | --- |
| **Address of Residence (street, nº, district and municipality):** |  |
| **Telephone contact:** | ( ) ________________________  ( ) ________________________  ( ) ________________________ |

| **Date of birth:** | **____/____/________** | **Date of death/cure:** | **____/_____/_________** |
| --- | --- | --- | --- |

1. **History of Prior Diseases:**

| **(1) Obesity:** | 1 – Yes | 2- No | 3- Unknown |  |
| --- | --- | --- | --- | --- |
| **(2) Weight:** | ___ ___ ___ Kg. and ___ ___ ___ gr | | | |
| **(3) Height:** | ___ m and ___ ___ cm | | | |
| **(4) BMI:** | _______________________________________ | | | |

| **(5)** **Cardiovascular diseases:** | 1 – Yes | 2- No | 3- Unknown |  |
| --- | --- | --- | --- | --- |

| **(6) Systemic arterial hypertension (SAH):** | 1 – Yes | 2- No | 3- Unknown |  |
| --- | --- | --- | --- | --- |
| **(7)** Regular use of medication: | 1 – Yes | 2- No | 3- Unknown |  |

| **(8) Coronary disease:** | 1 – Yes | 2- No | 3- Unknown |  |
| --- | --- | --- | --- | --- |
| **(9)** Regular use of medication: | 1 – Yes | 2- No | 3- Unknown |  |

| **(10) Cerebrovascular disease:** | 1 – Yes | 2- No | 3- Unknown |  |
| --- | --- | --- | --- | --- |
| **(11)** Regular use of medication: | 1 – Yes | 2- No | 3- Unknown |  |

| **(12) Congestive heart failure:** | 1 – Yes | 2- No | 3- Unknown |  |
| --- | --- | --- | --- | --- |
| **(13)** Regular use of medication: | 1 – Yes | 2- No | 3- Unknown |  |

| **(14) Diabetes mellitus:** | 1 – Yes | 2- No | 3- Unknown |  |
| --- | --- | --- | --- | --- |
| **(15)** Regular use of medication: | 1 – Yes | 2- No | 3- Unknown |  |

| **(16) Thyroid Disease:** | 1 – Yes | 2- No | 3- Unknown |  |
| --- | --- | --- | --- | --- |
| **(17)** Regular use of medication: | 1 – Yes | 2- No | 3- Unknown |  |

| **(18)**  **Respiratory Tract Diseases:** | 1 – Yes | 2- No | 3- Unknown |  |
| --- | --- | --- | --- | --- |

| **(19)** **Asthma:** | 1 – Yes | 2- No | 3- Unknown |  |
| --- | --- | --- | --- | --- |
| **(20)** Regular use of medication: | 1 – Yes | 2- No | 3- Unknown |  |

| **(21)** **COPD (Bronchitis or Pulmonary Emphysema):** | 1 – Yes | 2- No | 3- Unknown |  |
| --- | --- | --- | --- | --- |
| **(22)** Regular use of medication: | 1 – Yes | 2- No | 3- Unknown |  |

| **(23)** **Tuberculosis:** | 1 – Yes | 2- No | 3- Unknown |  |
| --- | --- | --- | --- | --- |
| **(24)** Regular use of medication: | 1 – Yes | 2- No | 3- Unknown |  |

| **(25)** **Cystic fibrosis:** | 1 – Yes | 2- No | 3- Unknown |  |
| --- | --- | --- | --- | --- |
| **(26)** Regular use of medication: | 1 – Yes | 2- No | 3- Unknown |  |

| **(27) Liver diseases:** | 1 – Yes | 2- No | 3- Unknown |  |
| --- | --- | --- | --- | --- |

| **(28)** **Acute:** | 1 – Yes | 2- No | 3- Unknown |  |
| --- | --- | --- | --- | --- |
| **(29)** If Yes, please specify: | | | |  |

| **(30)** **Chronic:** | 1 – Yes | 2- No | 3- Unknown |  |
| --- | --- | --- | --- | --- |
| **(31)** If Yes, please specify: | | | |  |

| **(32)** Regular use of medication: | 1 – Yes | 2- No | 3- Unknown |  |
| --- | --- | --- | --- | --- |

| **(33)**  **Hemoglobinopathy (Blood Disease):** | 1 – Yes | 2- No | 3- Unknown |  |
| --- | --- | --- | --- | --- |
| **(34)** If Yes, please specify: | | | |  |

| **(35)** Regular use of medication: | 1 – Yes | 2- No | 3- Unknown |  |
| --- | --- | --- | --- | --- |

| **(36)** **Autoimmune diseases (AID):** | 1 – Yes | 2- No | 3- Unknown |  |
| --- | --- | --- | --- | --- |

| **(37)** **Systemic lupus erythematosus (SLE):** | 1 – Yes | 2- No | 3- Unknown |  |
| --- | --- | --- | --- | --- |
| **(38)** Treatment: | 1 – Yes | 2- No | 3- Unknown |  |

| **(39)** **Rheumatoid arthritis:** | 1 – Yes | 2- No | 3- Unknown |  |
| --- | --- | --- | --- | --- |
| **(40)** Treatment: | 1 – Yes | 2- No | 3- Unknown |  |

| **(41)** **Other AIDs:** | 1 – Yes | 2- No | 3- Unknown |  |
| --- | --- | --- | --- | --- |
| **(42)** If Yes, please specify: | | | |  |
| **(43)** Treatment: | 1 – Yes | 2- No | 3- Unknown |  |

| **(44)**  **Other diseases (of any organ or system):** | 1 – Yes | 2- No | 3- Unknown |  |
| --- | --- | --- | --- | --- |
| **(45)**  If Yes, please specify: | | | |  |
| **(46)** Regular use of medication: | 1 – Yes | 2- No | 3- Unknown |  |

**(B) Immunodepression:**

| **(47)** **HIV:** | 1 – Yes | 2- No | 3- Unknown |  |
| --- | --- | --- | --- | --- |
| **(48)** **Aids:** | 1 – Yes | 2- No | 3- Unknown |  |
| **(49)** Date of last CD4: | ______/______/__________ | | |  |
| **(50)** Value of last CD4: |  | | |  |
| **(51)** Regular use of antiretrovirals (ARVs): | 1 – Yes | 2- No | 3- Unknown |  |

| **(52)** **Organ transplantation:** | 1 – Yes | 2- No | 3- Unknown |  |
| --- | --- | --- | --- | --- |
| **(53)** If Yes, please specify: |  | | |  |
| **(54)** Date of transplant: | ______/______/_________ | | |  |

| **(55)** **Neoplasms:** | 1 – Yes | 2- No | 3- Unknown |  |
| --- | --- | --- | --- | --- |
| **(56)** If Yes, which: |  | | |  |
| **(57)** Under treatment with chemotherapy: | 1 – Yes | 2- No | 3- Unknown |  |
| **(58)** Under treatment with radiotherapy: | 1 – Yes | 2- No | 3- Unknown |  |

| **(59)**  **Immunosuppressive medication:** | 1 – Yes | 2- No | 3- Unknown |  |
| --- | --- | --- | --- | --- |
| **(60)** Continuous use of corticosteroids: | 1 – Yes | 2- No | 3- Unknown |  |
| **(61)** For how long? | __________ months | | |  |
| **(62)**  If Yes, please specify: | | | |  |
| **(63)** Daily dose | __________mg | | |  |

**C) Current Health Care:**

| **(64)** Transferred from another service for hospitalization | 1 – Yes | 2- No | 3- Unknown |  |
| --- | --- | --- | --- | --- |
| **(65)** Current point of care location: |  | | |  |
| **(66)** Municipal: |  | | |  |
| **(67)** Date of Admission to Current Service : | ______/ _____/ ______ | | |  |
| **(68)** Medical Record Number: |  | | |  |

| **(69)** Total hospitalization time - PS. | _________ days |  |
| --- | --- | --- |
| **(70)** Date of Admission: | _____/ ______/_____ |  |
| **(71)** Time: | ___________________ |  |

| **(72)** Total hospitalization time - Infirmary. | _________ days |  |
| --- | --- | --- |
| **(73)**  Date of Admission: | _____/ ______/______ |  |
| **(74)** Time: | ___________________ |  |

| **(75)** Total hospitalization time - ICU. | _________ days |  |
| --- | --- | --- |
| **(76)** Date of Admission: | _____/ ______/______ |  |
| **(77) Time**: | ___________________ |  |

| **D)**  **Symptoms Reported on Hospital Admission** | | | | |
| --- | --- | --- | --- | --- |
| **(78)**  Date of onset of symptoms: | _____/ ______/______ | | |  |
| **(79)** Fever: | 1 – Yes | 2- No | 3- Unknown |  |
| **(80)** Maximum temperature: | _________º.C | | |  |
| **(81)** With this temperature was the patient using antipyretics? | 1 – Yes | 2- No | 3- Unknown |  |
| **(82)** Cough: | 1 – Yes | 2- No | 3- Unknown |  |
| **(83)** Headache: |  |  |  |  |
| **(84)** Chill: |  |  |  |  |
| **(85-A)** Dyspnea: |  |  |  |  |
| **(85-B)** Date of onset of dyspnea: | _____/ ______/______ | | |  |
| **(86)** Sore throat: |  |  |  |  |
| **(87)** Arthralgia: |  |  |  |  |
| **(88)** Myalgia: |  |  |  |  |
| **(89)** Conjunctivitis: |  |  |  |  |
| **(90)** Coryza: |  |  |  |  |
| **(91)** Diarrhea: |  |  |  |  |
| **(92)** Vomiting: |  |  |  |  |
| **(93)** Convulsions: |  |  |  |  |
| **(94)** Asthenia: |  |  |  |  |
| **(95)** Inappetence: |  |  |  |  |
| **(96)** Irritability: |  |  |  |  |
| **(97)** Others: |  |  |  |  |
| **(98)** Please specify other symptoms: |  | | |  |
| (99) Diagnostic hypotheses on admission: |  | | |  |

**E) Predictors of Severity in Adults (on admission)**

***WHEN ≤ 13 YEARS, JUMP TO ITEM (F)***

| **(100)**  **When information was not present on admission, note information obtained up to 24 hours after and, in this situation, enter date:** | | **_____/_____/_________.** | |  |
| --- | --- | --- | --- | --- |
| **(101)** Systolic Blood Pressure: | ___________________ | | |  |
| **(102)** Diastolic Blood Pressure: | ___________________ | | |  |
| **(103)** Heart Rate: | ___________________ | | |  |
| **(104)** Respiratory frequency: | ___________________ | | |  |
| **(105)** Axillary Temperature: | _________º.C | | |  |
| **(106)** With this temperature was the patient using antipyretics? | 1 – Yes | 2- No | 3- Unknown |  |
| **(107)** Mental confusion: | 1 – Yes | 2- No | 3- Unknown |  |
| **(108)** Dehydration: | 1 – Yes | 2- No | 3- Unknown |  |
| **(109)** Toxemia: | 1 – Yes | 2- No | 3- Unknown |  |
| **(110)** Cyanosis: | 1 – Yes | 2- No | 3- Unknown |  |
| **(111)** General condition compromised: | 1 – Yes | 2- No | 3- Unknown |  |

| **(112)**  **Ramsay Sedation Scale** | 1 – Yes | 2- No |  |
| --- | --- | --- | --- |
| **(113)** Value according to the Ramsay scale: | ___________________ | |  |
| **(114)** Date: | **_____/_____/_________.** | |  |

| **(115)**  **APACHE II Mortality Scale** | 1 – Yes | 2- No |  |
| --- | --- | --- | --- |
| **(116)**  Value according to the Apache II scale: | ___________________ | |  |
| **(117)** Date: | **_____/_____/_________.** | |  |

**F) Predictors of Severity in Children (≤) 13 Years (on admission)**

***WHEN ADULT, JUMP TO ITEM (G)***

| **(118) When information was not present on admission, note information obtained up to 24 hours after and, in this situation, enter date:** | | **_____/_____/_________.** | |  |
| --- | --- | --- | --- | --- |
| **(119)** Systolic Blood Pressure: | ___________________ | | |  |
| **(120)**  Diastolic Blood Pressure: | ___________________ | | |  |
| **(121)** Heart Rate: | ___________________ | | |  |
| **(122)** Respiratory frequency: | ___________________ | | |  |
| **(123)** Axillary Temperature: | _________º.C | | |  |
| **(124)** With this temperature was the patient using antipyretics? | 1 – Yes | 2- No | 3- Unknown |  |
| **(125)** Nasal flaring | 1 – Yes | 2- No | 3- Unknown |  |
| **(126)** Intercostal circulation: | 1 – Yes | 2- No | 3- Unknown |  |
| **(127)** Dehydration: | 1 – Yes | 2- No | 3- Unknown |  |
| **(128)** Toxemia: | 1 – Yes | 2- No | 3- Unknown |  |
| **(129)** Cyanosis: | 1 – Yes | 2- No | 3- Unknown |  |
| **(130)** Inappetence: | 1 – Yes | 2- No | 3- Unknown |  |
| **(131)** Vomiting: | 1 – Yes | 2- No | 3- Unknown |  |
| **(132)** Difficulty in drinking liquids: | 1 – Yes | 2- No | 3- Unknown |  |
| **(133)** General condition compromised: | 1 – Yes | 2- No | 3- Unknown |  |

| **(134) Ramsay Sedation Scale** | 1 – Yes | 2- No |  |
| --- | --- | --- | --- |
| **(135)** Value according to the Ramsay scale: | ___________________ | |  |
| **(136)** Date: | **_____/_____/_________.** | |  |

| **(137) APACHE II Mortality Scale** | 1 – Yes | 2- No |  |
| --- | --- | --- | --- |
| **(138)** Value according to the Apache II scale: | ___________________ | |  |
| **(139)** Date: | **_____/_____/_________.** | |  |

**G) Hospitalization: *(information regarding the current hospitalization)***

| **(140)** Using any antibiotics? | 1 – Yes | 2- No | 3- Unknown |  |
| --- | --- | --- | --- | --- |
| **(141)** Number of antibiotics used:  ***(For information on antibiotics use Annex 2)*** | | | _____________ |  |
| **(142)** Oxygen therapy: | 1 – Yes | 2- No | 3- Unknown |  |
| **(143)** Non-invasive mechanical ventilation (NIV): | 1 – Yes | 2- No | 3- Unknown |  |

| **(144)** Invasive mechanical ventilation (IV): | 1 – Yes | 2- No (Jump to 152) | 3- Unknown (Jump to 152) |  |
| --- | --- | --- | --- | --- |
| **(145)** Need for Mechanical Ventilation in the first 24 hours of admission: | 1 – Yes | 2- No | 3- Unknown |  |
| **(146)** Start date: | ____/_____/________ | | |  |
| **(147)** Duration: | ____________ days | | |  |

**Ventilation Mode:**

| **(148)** Synchronized Intermittent Mandatory Ventilation (SIMV): | 1 – Yes | 2- No | 3- Unknown |  |
| --- | --- | --- | --- | --- |
| **(149)** Controlled Ventilation (* PVC or ** VCV): |  |  |  |  |
| **(150)**  Ventilation with Frequency Oscillatory Increase: |  |  |  |  |
| **(151)** Others. Please specify: |  | | |  |
| (* PVC = Pressure controlled ventilation ** VCV = Volume controlled ventilation) | | | |  |

| **(152)**  **Received respiratory physiotherapy?** | 1 – Yes | 2- No (Jump to 156) | 3- Unknown (Jump to 156) |  |
| --- | --- | --- | --- | --- |
| **(**153) How many times/day? | ___________________ | | |  |
| **(154)** Start date: | **_____/_____/_________.** | | |  |
| **(155)** For how many days? | ___________________ | | |  |

| **(156)** Anticoagulant: | 1 – Yes | 2- No (Jump to 159) | 3- Unknown (Jump to 159) |  |
| --- | --- | --- | --- | --- |
| **(157)** Start date: | **_____/_____/_________.** | | |  |
| **(158)** For how many days? | ___________________ | | |  |

| **(159)** Gastro/enteral catheter: | 1 – Yes | 2- No (Jump to 162) | 3- Unknown (Jump to 162) |  |
| --- | --- | --- | --- | --- |
| **(160)** Start date: | **_____/_____/_________.** | | |  |
| **(161)** For how many days? | ___________________ | | |  |

| **(162)** Central venous catheter: | 1 – Yes | 2- No (Jump to 165) | 3- Unknown (Jump to 165) |  |
| --- | --- | --- | --- | --- |
| **(163)** Start date: | **_____/_____/_________.** | | |  |
| **(164)** For how many days? | ___________________ | | |  |

| **(165)** Tracheostomy: | 1 – Yes | 2- No (Jump to 168) | 3- Unknown (Jump to 168) |  |
| --- | --- | --- | --- | --- |
| **(166)** Start date: | **_____/_____/_________.** | | |  |
| **(167)** For how many days? | ___________________ | | |  |

| **(168)** Dialysis performed: | 1 – Yes | 2- No (Jump to 171) | 3- Unknown (Jump to 171) |  |
| --- | --- | --- | --- | --- |
| **(169)** Start date: | **_____/_____/_________.** | | |  |
| **(170)** For how many days? | ___________________ | | |  |

| **(171)** Received blood products: | 1 – Yes | 2- No (Jump to 174) | 3- Unknown (Jump to 174) |  |
| --- | --- | --- | --- | --- |
| **(172)** Start date: | **_____/_____/_________.** | | |  |
| **(173)** For how many days? | ___________________ | | |  |

| **(174)**  Chest drain: | 1 – Yes | 2- No (Jump to 177) | 3- Unknown (Jump to 177) |  |
| --- | --- | --- | --- | --- |
| **(175)** Start date: | **_____/_____/_________.** | | |  |
| **(176)** For how many days? | ___________________ | | |  |

| **(177) Admitted to the Intensive Care Unit?** | 1 – Yes | 2- No (Jump to 217) |  |
| --- | --- | --- | --- |
| **(178)**  **Date of ICU admission:** | **_____/_____/_________.** | |  |

**H) Predictors of Severity in Adults (admission to the ICU)**

***WHEN ≤ 13 YEARS, JUMP TO ITEM (I)***

| **(179)** Systolic Blood Pressure: | ___________________ | | |  |
| --- | --- | --- | --- | --- |
| **(180)** Diastolic Blood Pressure: | ___________________ | | |  |
| **(181)** Heart Rate: | ___________________ | | |  |
| **(182)** Respiratory frequency : | ___________________ | | |  |
| **(183)** Axillary Temperature: | _________º.C | | |  |
| **(184)** With this temperature was the patient using antipyretics? | 1 – Yes | 2- No | 3- Unknown |  |
| **(185)** Mental confusion: | 1 – Yes | 2- No | 3- Unknown |  |
| **(186)** Dehydration: | 1 – Yes | 2- No | 3- Unknown |  |
| **(187)** Toxemia: | 1 – Yes | 2- No | 3- Unknown |  |
| **(188)** Cyanosis: | 1 – Yes | 2- No | 3- Unknown |  |
| **(189)** General condition compromised: | 1 – Yes | 2- No | 3- Unknown |  |

| **(190)** **Ramsay Sedation Scale** | 1 – Yes | 2- No |  |
| --- | --- | --- | --- |
| **(191)** Value according to the Ramsay scale: | ___________________ | |  |
| **(192)** Date: | **_____/_____/_________.** | |  |

| **(193) APACHE II Mortality Scale** | 1 – Yes | 2- No |  |
| --- | --- | --- | --- |
| **(194)** Value according to the Apache II scale: | ___________________ | |  |
| **(195)** Date: | **_____/_____/_________.** | |  |

**I) Predictors of Severity in Children (≤) 13 Years (admission to ICU)**

***WHEN ADULT, JUMP TO ITEM (J)***

| **(196)** Systolic Blood Pressure: | ___________________ | | |  |
| --- | --- | --- | --- | --- |
| **(197)** Diastolic Blood Pressure: | ___________________ | | |  |
| **(198)** Heart Rate: | ___________________ | | |  |
| **(199)** Respiratory frequency: | ___________________ | | |  |
| **(200)** Axillary Temperature: | _________º.C | | |  |
| **(201)**  With this temperature was the patient using antipyretics? | 1 – Yes | 2- No | 3- Unknown |  |
| **(202)** Vacillation of the wings of the nose | 1 – Yes | 2- No | 3- Unknown |  |
| **(203)** Intercostal circulation: | 1 – Yes | 2- No | 3- Unknown |  |
| **(204)** Dehydration: | 1 – Yes | 2- No | 3- Unknown |  |
| **(205)** Toxemia: | 1 – Yes | 2- No | 3- Unknown |  |
| **(206)** Cyanosis: | 1 – Yes | 2- No | 3- Unknown |  |
| **(207)** Inappetence: | 1 – Yes | 2- No | 3- Unknown |  |
| **(208)** Vomiting: | 1 – Yes | 2- No | 3- Unknown |  |
| **(209)** Difficulty in drinking liquids: | 1 – Yes | 2- No | 3- Unknown |  |
| **(210)** General condition compromised: | 1 – Yes | 2- No | 3- Unknown |  |

| **(211) Ramsay Sedation Scale** | 1 – Yes | 2- No |  |
| --- | --- | --- | --- |
| **(212)**  Value according to the Ramsay scale: | ___________________ | |  |
| **(213)** Date: | **_____/_____/_________.** | |  |

| **(214) APACHE II Mortality Scale** | 1 – Yes | 2- No |  |
| --- | --- | --- | --- |
| **(215)**  Value according to the Apache II scale: | ___________________ | |  |
| **(216)** Date: | **_____/_____/_________.** | |  |

**J) Use of Oseltamivir:**

| **(217)** Oseltamivir: | 1 – Yes | 2- No  (Jump to 229) | 3- Unknown  (Jump to 229) |  |
| --- | --- | --- | --- | --- |

| **(218)** Start date: | **_____/_____/_________.** | | |  |
| --- | --- | --- | --- | --- |
| **(219)** Dose: | _________ (mg) 12/12h | | |  |
| **(220)** Total number of days of use: | ___________________ days | | |  |
| **(221)** Was the Oseltamivir treatment regimen changed at any time? | 1 – Yes | 2- No  (Jump to 227) | 3- Unknown  (Jump to 227) |  |

| **(222)** Date of change: | **_____/_____/_________.** |  |
| --- | --- | --- |
| **(223)** New dose: | _________ (mg) |  |
| **(224)** Number of times per day: | ___________________ |  |
| **(225)** Reason for change: | 1 – Obesity (Jump to 227) |  |
|  | 2 - Use of gastro/enteral catheter (Jump to 227) |  |
|  | 3 - Renal insufficiency (Jump to 227) |  |
|  | 4 – SARS (Jump to 227) |  |
|  | 5 - Newborn (Jump to 227) |  |
|  | 6 - Others |  |
| **(226)** What other reasons? |  |  |

| **(227)** Is there a record of suspected adverse reaction/event to Oseltamivir? | 1 – Yes | 2- No  (Jump to 229) | 3- Unknown  (Jump to 229) |  |
| --- | --- | --- | --- | --- |
| **(228)** What reactions? |  | | |  |

**K) Complications:**

| **(229)**  **Were there any complications (described in the medical chart) during the period of hospitalization?** | 1 – Yes | 2- No  (Jump to 256) |  |
| --- | --- | --- | --- |

**Event**

| **(230-A) SHOCK** | 1 – Yes | 2- No |  |
| --- | --- | --- | --- |
| **(230-B)** Start Date | **_____/_____/_________.** | |  |

| **(231-A) SEPSIS** | 1 – Yes | 2- No |  |
| --- | --- | --- | --- |
| **(231-B)** Start Date | **_____/_____/_________.** | |  |

| **(232-A)** **COAGULATION DISTURBANCE** | 1 – Yes | 2- No |  |
| --- | --- | --- | --- |
| **(232-B)** Start Date | **_____/_____/_________.** | |  |

| **(233- A)** **PULMONARY** | 1 – Yes | 2- No |  |
| --- | --- | --- | --- |
| **(233- B)** Start Date | **_____/_____/_________.** | |  |

| **(234-A)** Severe Acute Respiratory Syndrome (SARS) | 1 – Yes | 2- No |  |
| --- | --- | --- | --- |
| **(234-B)** Start Date | **_____/_____/_________.** | |  |

| **(235-A)** Pleural effusion | 1 – Yes | 2- No |  |
| --- | --- | --- | --- |
| **(235-B)** Start Date | **_____/_____/_________.** | |  |

| **(236-A)** Pulmonary embolism | 1 – Yes | 2- No |  |
| --- | --- | --- | --- |
| **(236-B)** Start Date | **_____/_____/_________.** | |  |

| **(237-A)** Pulmonary hemorrhage | 1 – Yes | 2- No |  |
| --- | --- | --- | --- |
| **(237-B)** Start Date | **_____/_____/_________.** | |  |

| **(238-A)** Other pulmonary | 1 – Yes | 2- No |  |
| --- | --- | --- | --- |
| **(238-B)** Start Date | **_____/_____/_________.** | |  |

| **(239-A) CARDIOVASCULAR DISEASE** | 1 – Yes | 2- No |  |
| --- | --- | --- | --- |
| **(239-B)** Start Date | **_____/_____/_________.** | |  |

| **(240-A)** Cardiac insufficiency | 1 – Yes | 2- No |  |
| --- | --- | --- | --- |
| **(240-B)** Start Date | **_____/_____/_________.** | |  |

| **(241-A)** Acute myocardial infarction | 1 – Yes | 2- No |  |
| --- | --- | --- | --- |
| **(241-B)** Start Date | **_____/_____/_________.** | |  |

| **(242-A)** Atrioventricular Block (1st, 2nd or 3rd). | 1 – Yes | 2- No |  |
| --- | --- | --- | --- |
| **(242-B)** Start Date | **_____/_____/_________.** | |  |

| **(243-A)** Supraventricular or Ventricular Tachycardia (Arrhythmia) | 1 – Yes | 2- No |  |
| --- | --- | --- | --- |
| **(243-B)** Start Date | **_____/_____/_________.** | |  |

| **(244-A)** Endocarditis | 1 – Yes | 2- No |  |
| --- | --- | --- | --- |
| **(244-B)** Start Date | **_____/_____/_________.** | |  |

| **(245-A)** Myocarditis | 1 – Yes | 2- No |  |
| --- | --- | --- | --- |
| **(245-B)** Start Date | **_____/_____/_________.** | |  |

| **(246-A)** Pericarditis | 1 – Yes | 2- No |  |
| --- | --- | --- | --- |
| **(246-B)** Start Date | **_____/_____/_________.** | |  |

| **(247-A)** Pericardial tamponade | 1 – Yes | 2- No |  |
| --- | --- | --- | --- |
| **(247-B)** Start Date | **_____/_____/_________.** | |  |

| **(248-A)** Cardiopulmonary arrest | 1 – Yes | 2- No |  |
| --- | --- | --- | --- |
| **(248-B)** Start Date | **_____/_____/_________.** | |  |

| **(249-A) GASTROINTESTINAL** | 1 – Yes | 2- No |  |
| --- | --- | --- | --- |
| **(249-B)** Start Date | **_____/_____/_________.** | |  |

| **(250-A) HEPATIC** | 1 – Yes | 2- No |  |
| --- | --- | --- | --- |
| **(250-B)** Start Date | **_____/_____/_________.** | |  |

| **(251-A) RENAL** | 1 – Yes | 2- No |  |
| --- | --- | --- | --- |
| **(251-B)** Start Date | **_____/_____/_________.** | |  |

| **(252-A)** **NEUROLOGICAL** | 1 – Yes | 2- No |  |
| --- | --- | --- | --- |
| **(252-B)** Start Date | **_____/_____/_________.** | |  |

| **(253-A) INFECTION** | 1 – Yes | 2- No |  |
| --- | --- | --- | --- |
| **(253-B)** Start Date | **_____/_____/_________.** | |  |

| **(254-A)** **METABOLIC** | 1 – Yes | 2- No |  |
| --- | --- | --- | --- |
| **(254-B)** Start Date | **_____/_____/_________.** | |  |

| **(255-A) OTHERS. Please specify:**  ***(Note only if other complications are registered in the records -***  You ***should not interpret the complications).*** | 1 – Yes | 2- No |  |
| --- | --- | --- | --- |
| **(255-B)** Start Date | **_____/_____/_________.** | |  |

**L) Examination results**

| **EXAMINATIONS** | **FIRST**  **RESULT**  **DATE__/____/____** | **FIRST**  **RESULT**  **(Values)** | **MOST ALTERED RESULT**  **DATE__/____/____** | **MOST ALTERED RESULT**  **(Values)** |
| --- | --- | --- | --- | --- |
| **(256)** Hemoglobin (g/dL | ___/____/_____ |  | ___/____/_____ |  |
| **(257)** Hematocrit (%) | ___/____/_____ |  | ___/____/_____ |  |
| **(258)** Leukocytes (mm^3^) | ___/____/_____ |  | ___/____/_____ |  |
| **(259)** Rods (%) |  |  |
| **(260)** Segmented (%) |  |  |
| **(261)** Lymphocytes (%) |  |  |
| **(262)** Platelets (mm^3^ ) | ___/____/_____ |  | ___/____/_____ |  |
| **(263)** Glycemia (mg/dL) | ___/____/_____ |  | ___/____/_____ |  |
| **(264)** C reactive protein | ___/____/_____ |  | ___/____/_____ |  |
| **(265)**  Creatine phosphokinase | ___/____/_____ |  | ___/____/_____ |  |
| **(266)** LDH | ___/____/_____ |  | ___/____/_____ |  |
| **(267)**  Indirect Bilirubin | ___/____/_____ |  | ___/____/_____ |  |
| **(268)** Direct Bilirubin | ___/____/_____ |  | ___/____/_____ |  |
| **(269)** Glutamic-oxalacetic transaminase | ___/____/_____ |  | ___/____/_____ |  |
| **(270)** Pyruvic glutamic transaminase | ___/____/_____ |  | ___/____/_____ |  |
| **(271)** Sodium | ___/____/_____ |  | ___/____/_____ |  |
| **(272)** Potassium | ___/____/_____ |  | ___/____/_____ |  |
| **(273)**  Urea | ___/____/_____ |  | ___/____/_____ |  |
| **(274)** Creatinine | ___/____/_____ |  | ___/____/_____ |  |
| **(275)** Alkaline phosphatase | ___/____/_____ |  | ___/____/_____ |  |
| **(276)** GT Range | ___/____/_____ |  | ___/____/_____ |  |
| **(277)** Albumin | ___/____/_____ |  | ___/____/_____ |  |
| **(278)** Globulin | ___/____/_____ |  | ___/____/_____ |  |
| **(279)** Prothrombin Time (INR) | ___/____/_____ |  | ___/____/_____ |  |
| **(280)**  Activated thromboplastin time | ___/____/_____ |  | ___/____/_____ |  |

| **(281)** Chest radiography performed | 1 – Yes | 2- No (Jump to 286) |  |
| --- | --- | --- | --- |
| **(282)** Report in the medical record: | 1 – Yes | 2- No |  |
| **(283)** Only description without report: | 1 – Yes | 2- No |  |
| **(284)** Number of chest XRs performed: | ___________________ | |  |
| **(285-A)**  First Result: | **_____/_____/_________.** | |  |
| **(285-B)** Result | 1 – Alveolar | |  |
|  | 2 – Interstitial | |  |
|  | 3 - Alveolar + Interstitial | |  |
|  | 4 - Consolidated | |  |
|  | 5 – Pleural effusion | |  |
|  | 6 – Others: _____________________________________ | |  |
|  | 7 - Unknown | |  |
| **(285-C)** Most altered result | **_____/_____/_________.** | |  |
| **(285-D)** Result | 1 – Alveolar | |  |
|  | 2 – Interstitial | |  |
|  | 3 - Alveolar + Interstitial | |  |
|  | 4 - Consolidated | |  |
|  | 5 – Pleural effusion | |  |
|  | 6 – Others: _____________________________________ | |  |
|  | 7 - Unknown | |  |

***M) Microbiology (fill in only positive results)***

| **Laboratory method used** | **Isolation was obtained from samples of** | **Pathogen identified** | **Date of collection**  **(most important first result)** |
| --- | --- | --- | --- |
| [ ] PCR  [ ] Latex  [ ] Counter Immunoelectrophoresis  [ ] Others  Which____________ | **(286)** Blood  [ ] Yes [ ] No [ ] Unknown  **(287)** Líquor  [ ] Yes [ ] No [ ] Unknown  **(288)** Tracheal aspirate  [ ] Yes [ ] No [ ] Unknown  **(289)** Bronchoalveolar lavage  [ ] Yes [ ] No [ ] Unknown  **(290)** Pleural fluid  [ ] Yes [ ] No [ ] Unknown  **(291)** Others _______________ | [ ] *Streptococcus pneumoniae*  [ ] *Group A Streptococcus*  [ ] *Haemophilus influenzae*  [ ] *Staphylococcus aureus*  [ ] *Neisseria meningitidis* (specify serogroup, if known)*:___________*  [ ] Others  WHICH?__________________________ | _____/____/________ |
| [ ] Culture  [ ] PCR  [ ] Latex  [ ] Counter Immunoelectrophoresis  [ ] Others  Which____________ | **(292)** Blood  [ ] Yes [ ] No [ ] Unknown  **(293)** Líquor  [ ] Yes [ ] No [ ] Unknown  **(294)** Tracheal aspirate  [ ] Yes [ ] No [ ] Unknown  **(295)** Bronchoalveolar lavage  [ ] Yes [ ] No [ ] Unknown  **(296)** Pleural fluid  [ ] Yes [ ] No [ ] Unknown  **(297)** Others _______________ | [ ] *Streptococcus pneumoniae*  [ ] *Group A Streptococcus*  [ ] *Haemophilus influenzae*  [ ] *Staphylococcus aureus*  [ ] *Neisseria meningitidis* ( specify serogroup, if known)*:___________*  [ ] Others  WHICH?__________________________ | _____/____/________ |

| **(298)** Number of exams performed: | ___________________ |  |
| --- | --- | --- |
| **(299)**  Number of negative exams: | ___________________ |  |
| **(300)** Number of positive exams: | ___________________ |  |

**N)**

| **(301)**  **rRT-PCR (real-time PCR) for Influenza A (H1N1) - *Report attached to medical record*** | 1 – positive | 2 - negative  (Jump to 304) | 3 – not found  (Jump to 304) |  |
| --- | --- | --- | --- | --- |
| **(302)** Date of collection: | **_____/_____/_________.** | | |  |
| **(303)** Sample Type: | Nasopharyngeal secretion | | |  |
|  | Bronchoalveolar lavage | | |  |
|  | Stool | | |  |
|  | Postmortem tissue | | |  |
|  | Serum | | |  |
|  | Other. Which:____________________________________ | | |  |

| **(304) Death** | 1 – Yes | 2- No  *If patient is pregnant, complete Annex 1.*  *If not pregnant, end.* |  |
| --- | --- | --- | --- |
| **(305) No. Of DC** | ___________________ | |  |

| **( (306)** **Causes of death recorded on the Death Certificate (DC)** | | **ICD** |
| --- | --- | --- |
| Part I | a) |  |
|  | b) |  |
|  | c) |  |
|  | d) |  |
| Part II |  |  |

| **(307) Was a Necropsy performed?** | 1 – Yes | 2 - No  (Jump to 314) | 3 - Unknown  (Jump to 314) |  |
| --- | --- | --- | --- | --- |

| **(308)** Reported cause of death: |  |  |
| --- | --- | --- |

| **(309)** Description of the necropsy report: |  |  |
| --- | --- | --- |

| **(310)** Macroscopic examination: |  |  |
| --- | --- | --- |

| **(311)** Microscopic examination: |  |  |
| --- | --- | --- |

| **(312)** rRT-PCR for FLU A (H1N1) from organs and tissues: | 1 – Yes | 2- No  (Jump to 314) | 3- Unknown  (Jump to 314) |  |
| --- | --- | --- | --- | --- |
| **(313)** Specify tissue(s) |  | | |  |

| **(314)** Date of death: | **_____/_____/_________.** |  |
| --- | --- | --- |

**ANNEX 1**

**P) PREGNANT WOMEN**

**History Related to the Pregnancy-Puerperal Cycle:**

**(316)** Prenatal follow-up location: _______________________________ **(317)** Number of prenatal consultations: ________

**(318)** LMP: _____/______/______ **(319)** Blood group: ABO____RH ____

**(320)** Isoimmunization case? [ ] yes [ ] no [ ] not known

**Obstetric Complications/Intercurrences:**

**(321)** Premature labor [ ] yes [ ] no [ ] not known

**(322)** Bleeding [ ] yes [ ] no [ ] not known

**(323)** Uterine atony [ ] yes [ ] no [ ] not known

**(324)** Placenta accreta [ ] yes [ ] no [ ] not known

**(325)** Puerperal infection [ ] yes [ ] no [ ] not known

**(326)** Embolisms [ ] yes [ ] no [ ] not known

**(327)** Deep vein thrombosis [ ] yes [ ] no [ ] not known

**(328)** Others [ ] yes [ ] no [ ] not known

**(329)** If yes, please specify: _______________________________________________________________

**Factors for Pregnancy Risk:**

**(330)** Multiple gestation: [ ] yes [ ] no [ ] not known

**(331)** Gestational diabetes: [ ] yes [ ] no [ ] not known

**(332)** Malformation: [ ] yes [ ] no [ ] not known

**(333)** PSHD: [ ] yes [ ] no [ ] not known

**(334)** Pre eclampsia: [ ] yes [ ] no [ ] not known

**(335)** Eclampsia: [ ] yes [ ] no [ ] not known

**(336)** Cardiopathies: [ ] yes [ ] no [ ] not known

**(337)** Urinary infection: [ ] yes [ ] no [ ] not known

**(338)** Bleeding: [ ] yes [ ] no [ ] not known. **(339)** If yes, specify trimester: _______

**(340)** Pregnancy anemia: [ ] yes [ ] no [ ] not known

**(341)** STD: [ ] yes [ ] no [ ] not known

**(342)** Others: [ ] yes [ ] no [ ] not known. **(343)** If yes: _______________________

**Obstetric Background:**

**(344)** Number of previous pregnancies? ______ **(345)** Live births: ______ **(346)** Fetal deaths: ______

**(347)** Miscarriages? [ ] yes [ ] no [ ] not known

**(348)** When was the last pregnancy (prior to this, in years)? _______

**(349)** Any health problems in previous pregnancies? [ ] yes [ ] no [ ] not known

**(350)** If yes, please specify: ______________________________________________________________________

**(351)** Did the delivery/miscarriage occur during hospitalization? [ ] yes [ ] no [ ] not known

**(352)** If yes, type of delivery: [ ] normal [ ] caesarian [ ] no delivery [ ] not known

**(353)** Date of delivery/miscarriage: ______/ ______/ ________ **(354)** Indication of labor? ______________________________

**(355)** Other procedures performed? [ ] yes [ ] no [ ] not known

**(356)** If yes: [ ] hysterectomy [ ] hypogastric artery ligation [ ] others (please specify) _______________

**P) Newborn Data:**

**(357)** Condition at birth: [ ] live [ ] dead [ ] not known

If live, **(358)** Evolution [ ] Discharged [ ] Death **(359)** If death, date _____/_______/_________

**(360)** Gestational Age (GA): _______ weeks

**(361)** Apgar 1^st^ minute______ **(362)** Apgar 5^th^ minute______

**(363)** Birth weight: _______(g)

**(364)** Inpatient Unit: [ ] Neonatal ICU [ ] Joint accommodation [ ] Nursery

**(365)** **Diagnostic hypotheses:**

[ ] Fetal macrosomia [ ] Malformation [ ] Suspected Congenital Infection [ ] Non-physiological jaundice

[ ] Peri natal asphyxia [ ] Congenital anomalies [ ] Acute anemia [ ] Hemorrhagic syndrome [ ] Convulsions

[ ] Umbilical cord prolapse [ ] Fetal distress [ ] Hyaline membrane [ ] Sepsis [ ] Hemolytic disease

**Q) Occurrence of Death in Pregnant Women:**

**(366)** **Date of death: _____/ _____/ ________**

**(367)** When? [ ] before the birth/miscarriage [ ] at the time of birth/miscarriage [ ] after the birth/miscarriage

**(368)** Sector of the hospital where the death occurred: [ ] ICU [ ] Infirmary [ ] Obstetric center [ ] Emergency room [ ] Not known [ ] Others **(369)** Describe: _______________________________________

**R) Data from the Puerperium**:

**(370)** Intercurrences in the puerperium: [ ] yes [ ] no [ ] not known

**(371)** If Yes, please specify:

[ ] Up to two hours after delivery [ ] More than two hours after delivery [ ] Late puerperium (more than 45 days)

[ ] Others, please specify:_________________________________________________________________________
